# Vaccine uptake and immune responses to HBV infection amongst vaccinated and non-vaccinated healthcare workers, household and sexual contacts to chronically infected HBV individuals in the South West Region of Cameroon

**DOI:** 10.1371/journal.pone.0200157

**Published:** 2018-07-16

**Authors:** Henry Dilonga Meriki, Kukwah Anthony Tufon, Damian Nota Anong, Nyeke James Tony, Tebit Emmanuel Kwenti, Ayah Flora Bolimo, Youmbi Sylvain Kouanou, Theresa Nkuo-Akenji

**Affiliations:** 1 Department of Microbiology and Parasitology, University of Buea, Buea, South West Region, Cameroon; 2 Buea Regional Hospital, Buea, Southwest Region, Cameroon; 3 Department of Public health and Hygiene, Faculty of Health science, University of Buea, Buea, Southwest Region, Cameroon; 4 Department of Biological Science, Faculty of Science, University of Bamenda, Bamenda, North West Region, Cameroon; 5 Department of Medical Laboratory Science, Faculty of Health science, University of Buea, Buea, Southwest Region, Cameroon; National Institutes of Health, UNITED STATES

## Abstract

**Background:**

HBV infection affects about 257 million people globally and Sub-Saharan Africa has the highest burden. The disease still constitutes a major public health problem despite the advent of preventive measures like the HBV vaccine. This study was aimed at identifying factors that influence vaccine uptake and the efficacy of administered vaccines among people at high risk of HBV infection.

**Methods:**

This was a cross-sectional study conducted between January 2016 and December 2017. A pretested semi-structured questionnaire was used to capture information on sociodemographic and vaccination status from healthcare workers, household and sexual contacts to HBV infected people. HBV serological panel as well as quantitative anti-HBs ELISA test was done for all participants. Additional information was obtained from the institutions that administered the vaccines.

**Results:**

A total of 265 participants with a mean age of 32.1±8.7 were enrolled. Eighty (30.2%) of them had received at least 1 dose of the HBV vaccine while 185 (69.8%) were unvaccinated. Healthcare workers were the most vaccinated (37%). Ignorance, negligence, fear of injection and the cost of the vaccine all contributed to poor vaccine uptake in the study population. Natural immunity was seen in 9 (3.4%) of the participants. Only 64.9% of the vaccinated participants attained the desirable level of anti-HBs (≥10mIU/ml) 1–2 months after ≥ 3 doses of the vaccine. Age, gender, obesity, alcohol and smoking were not significantly associated with poor immune responses. No standardized protocol was followed by the institutions administering the vaccine.

**Conclusion:**

This study revealed very poor vaccine uptake and poor immune responses to the HBV vaccine in the study population and this should urge the health sector in Cameroon to intensify their sensitization on HBV vaccine, standardize the protocol for storing and administering the vaccine, subsidize the cost of the vaccine especially amongst healthcare workers and encourage anti-HBs post vaccination testing.

## Introduction

An estimated 1 million people die each year from liver disease, including liver cancer and cirrhosis probably as a result of viral hepatitis like HBV infection [1]. Approximatively 257 million people are infected with HBV globally and most of these people reside in low-income and middle-income countries [2,3] particularly in Africa [4]. Cameroon happens to be one of the countries significantly affected with an HBV prevalence of 11.2% [5]. The disease still stands as a major public health problem despite the advent of preventive measures like the hepatitis B vaccine.

The vaccine is produced following the purification of HBsAg obtained from the plasma of persons with chronic HBV infection [6–8]. This vaccine is meant to prevent HBV infection and its associated liver complications [6]. To achieve this, the vaccine must trigger an immune response which would produce anti-HBs at a concentration of ≥10 mIU/mL at least 1 month and at most 2 months after the 3^rd^ dose [9,10]

Intramuscular administration of ≥3 doses of the vaccine at 0, 1, and 6 months [11] should trigger a protective antibody response in approximately 30%–55% of healthy adults ≤40 years of age after the first dose, 75% after the second dose and >90% after the third dose [12]. Other vaccine schedules which include 0, 1, and 4 months or 0, 2, and 4 months (Engerix vaccine brand) have shown similar results to those obtained on a 0-, 1-, 6-month schedule [13]. Increasing the interval between the first 2 doses has little effect on the final antibody concentration [14,15]. The third dose acts primarily as a booster [16]

About 5–15% of vaccinees may not develop the expected immune response following the complete dose administration of the vaccine. [17]. Usually, about 30–50% of people who do not respond to a primary 3-dose vaccine series with anti-HBs concentrations of ≥10 mIU/ml, may respond to an additional vaccine dose or to a 3-dose revaccination series [17,18]. Those who still do not respond to a second series of vaccination may respond to intradermal administration [19] or to a high dose vaccine [20] or to a double dose of a combined Hepatitis A and B vaccine [21]. For some reason, some people (usually referred to as non-responders) still do not respond even to a second series of the vaccine. The Center for Disease Control and Prevention (CDC) has outlined some recommendations that must be considered for HBV vaccine non-responders in order to limit their chances of contracting the infection. As such, the need to identify such people in a country with a high HBV endemicity especially from high risk groups cannot be overemphasized.

Some already identified factors like age, obesity, smoking, alcohol consumption, immunosuppression, genetic factors and comorbid conditions including diabetes may influence the expected immune response following vaccine administration [22–29]. For this reason, a post vaccine quantitative test for anti-HBs is usually recommended especially for high risk groups like neonates born to HBV carrier mothers, visitors to regions with high HBV endemicity immunocompromised people (e.g. people living with HIV and persons undergoing hemodialysis), healthcare workers, household and sexual contacts to HBV infected people [9,30].

In Cameroon, no test is usually done after vaccine completion to identify and measure anti HBs production. This implies that little or no measures are taken to control and confirm the effectiveness of the vaccine after administration despite the lack of proper storage facilities, limited competent personnel and constant power failure which could have a direct detrimental effect on vaccine potency. As one of the objectives of this study, we evaluated the antibody response to HBV vaccine (after complete administration of 3 doses) and tried to identify possible reasons of no or poor responses in our community.

Active immunity to HBV infection is either acquired artificially (vaccine) or naturally (resolved infection) and they can be differentiated with the presence of hepatitis B core antibody (anti-HBc-total) in the latter [30] indicating that the person must have been infected before. Detection and quantification of anti-HBs for both cases remain the sole determinant of immunity to HBV infection. The presence of anti-HBs following acute HBV infection basically indicates recovery and immunity against possible re-infection with the virus. However, the time of testing matters as anti HBs may not be detected in individuals with distantly resolved HBV infection and this is possibly because the anti-HBs levels at that time may have dropped below the testing method’s limit of detection. Such individuals may present with the following serologic results: HBsAg-negative, anti-HBs-negative and anti-HBc total-positive [31,32]. It is possible that some high-risk groups (health care workers, sexual and household contacts to chronic HBV patients etc) may fall in this category or may present with detectable anti-HBs as a result of previous contact with the virus which did not lead to chronic infection. As such, the use of HBsAg, anti-HBs and anti-HBc total as primary diagnostic tests for HBV infection [30] in high risk groups is important as this could give a thorough and more detailed information on their HBV status, help in understanding naturally acquired immune response to HBV infection, ascertain the need for the vaccine as well as identify individuals who are at risk of HBV reactivation following immunosuppression. Low levels of HBV viral particles remain in liver cells after recovery from acute hepatitis B. People who have been exposed to HBV are at risk of reactivation of hepatitis B infection if they become immunocompromised over time [33]. Reactivation of HBV can occur in both HBsAg positive and HBsAg negative but anti-HBc positive individuals in case of potent immunosuppression [34].

## Materials and methods

### Study design, site and population

This was a hospital-community based cross-sectional study that enrolled both HBV vaccinated and non-vaccinated but exposed individuals ≥ 18 years old. The study was conducted in the Buea Regional hospital in the South West region of Cameroon. We enrolled high-risk groups like healthcare workers from Buea Regional Hospital and sexual/household contacts of some chronically infected HBV patients. We considered a healthcare worker (HCW) to be any paid or unpaid person providing health care, or working or training in health-care settings, who have reasonably anticipated risks for exposure to infectious materials, including blood or body fluids [17]. A sexual partner (SP) was considered to be anybody who is or has been in a sexual relationship with a chronically infected HBV person for more than 6 months while a household contact (HHC) was considered to be anybody who has been living in the same house with a chronically infected HBV person for more than 6 months. The chronically infected HBV patients (who linked us to their sexual/household contacts) were people who had been earlier enrolled for another study following a free screening exercise and general consultation at the Buea Regional hospital.

### Ethical approval and consent to participate

This study was approved by the National Ethics Committee of Research for Human Health (NECRHH) in Cameroon and the administrative authorities of the hospitals concerned. Each participant signed a consent form before enrolment. A counsellor verbally explained the content of the consent form for those who could not read nor write. Consent was obtained from the parents or guardians of minors enrolled in the study.

### Sample size calculation

The sample size was estimated using the formula for sample size calculation described by Swinscow [35] as follows;

$$n=\frac{Z^{2}x\;p\;\left(1-p\right)}{e^{2}}$$

Z=1.96

P=Proportionofvaccinecoverageforhealthcareworkersinasimilarstudy=19%

[36]

e=errorrate=0.05

$$n=\frac{1.96x\;0.19\;(1-0.19)}{0.05^{2}}=236.5$$

We needed to recruit at least 237 participants for this study.

Chronic hepatitis B patients who consulted at the Buea Regional Hospital between January 2016 and December 2017 linked us to their sexual and household contacts who were enrolled in this study. The high prevalence (8.0%) of HBV infection in the South West Region of Cameroon [5] warrants the understanding and assessment of vaccine uptake amongst any household and/or sexual contact to HBV infected patients in order to improve on the control of the infection.

### Data collection

A standard pretested semi-structured questionnaire was administered (interview based) to all participants to assess their knowledge on HBV infection (nature of the disease, transmission routes, risk factors and HBV vaccine awareness). Participants were considered to have good knowledge if they were able to provide 70% or more of the correct responses [37]. The questionnaire also captured information on HBV vaccine status, risk factors for poor immune responses to the vaccine, previous contact with an HBV-infected person and nature of contact for in the past six months. For vaccinated participants, further information on the number of doses, dates received, brand of vaccine, volume received, lot number of vaccine was obtained from their vaccination cards and/or from the institution that administered the vaccines.

Information on vaccine administration protocol and storage was also obtained from the institutions administering the vaccines following a site visit.

Weight and height measurement was done for all vaccinated participants and this information was used to calculate body mass index (BMI) using the formula below:

$$BMI=weight(kg)÷Height(m^{2})$$

[38]

### Sample collection and analysis

A trained phlebotomist collected about 5 ml of blood from each participant in tubes with no anticoagulant. The samples were centrifuged at 1000g for 5 minutes to obtain sera. The sera were first of all screened for HIV and HCV antibodies using Abbot Determine and Acon^®^ Laboratories Inc. respectively. All samples were tested for the presence of HBsAg, anti-HBs and anti-HBc total qualitatively using Blue Cross Bio-Medical Co., Ltd and subjected to quantitative anti-HBs ELISA test with BIOELISA anti-HBs testing kit following manufacturer’s instructions. In order to get the anti-HBs concentrations (in mIU/ml) a calibration curve was generated using the negative control, low positive calibrator and high positive calibrator. A linear equation was generated from the calibration curve and used to calculate x (concentration) from all the corresponding absorbance (y) obtained.

### Statistical analysis

Data analysis was carried out using SPSS (Statistical Package for Social Sciences) version 21. Data were presented as number of cases, percentages and mean ± standard deviation. Categorical comparisons were performed using the Pearson’s Chi-square test or the Fisher’s exact test (for 2 by 2 cells having values < 5). Spearman correlation was used to assess anti-HBs decline over time. Adjusted odds ratio (OR) was performed for factors that recorded p value < 0.2 in the crude OR. A two-sided p value < 0.05 was considered significant for all analyses.

## Results

### General population description

A total of 265 participants with a mean age of 32.1±8.7 were enrolled. This population consisted of individuals vaccinated against HBV and non-vaccinated healthcare workers (HCW), household contacts (HHC) and sexual partners (SP) to HBV infected participants. The population was made up of 168 (63.4%) females and 97 (36.6) males. Age and gender distribution across the different groups of participants is presented in Table 1.

**Table 1:** Age and gender distribution across the different groups of participants.

Groups	n	Mean age ± SD (years)	Age range (years)	Gender	n (%)
Healthcare workers	127	33.3 ± 7.0	23–54	Female	92 (72.4)
				Male	35 (27.6)
Household contacts	52	23.0 ± 15.6	18–65	Female	29 (55.8)
				Male	23 (44.2)
Sexual partners	71	31.0 ± 6.7	19–45	Female	42 (59.2)
				Male	29 (40.9)
Others*	15	29.4 ± 6.1	19–37	Female	5 (33.3)
				Male	10 (66.7)

* Vaccinated individuals who are neither healthcare workers nor sexual/household contacts of HBV infected patients.

Forty-six (64.7%) of the sexual partners and 22 (42.3%) of the household contacts had basic knowledge (aware of the disease and its transmission routes) of HBV infection. All the Healthcare workers had knowledge of HBV infection.

**Fig 1:**
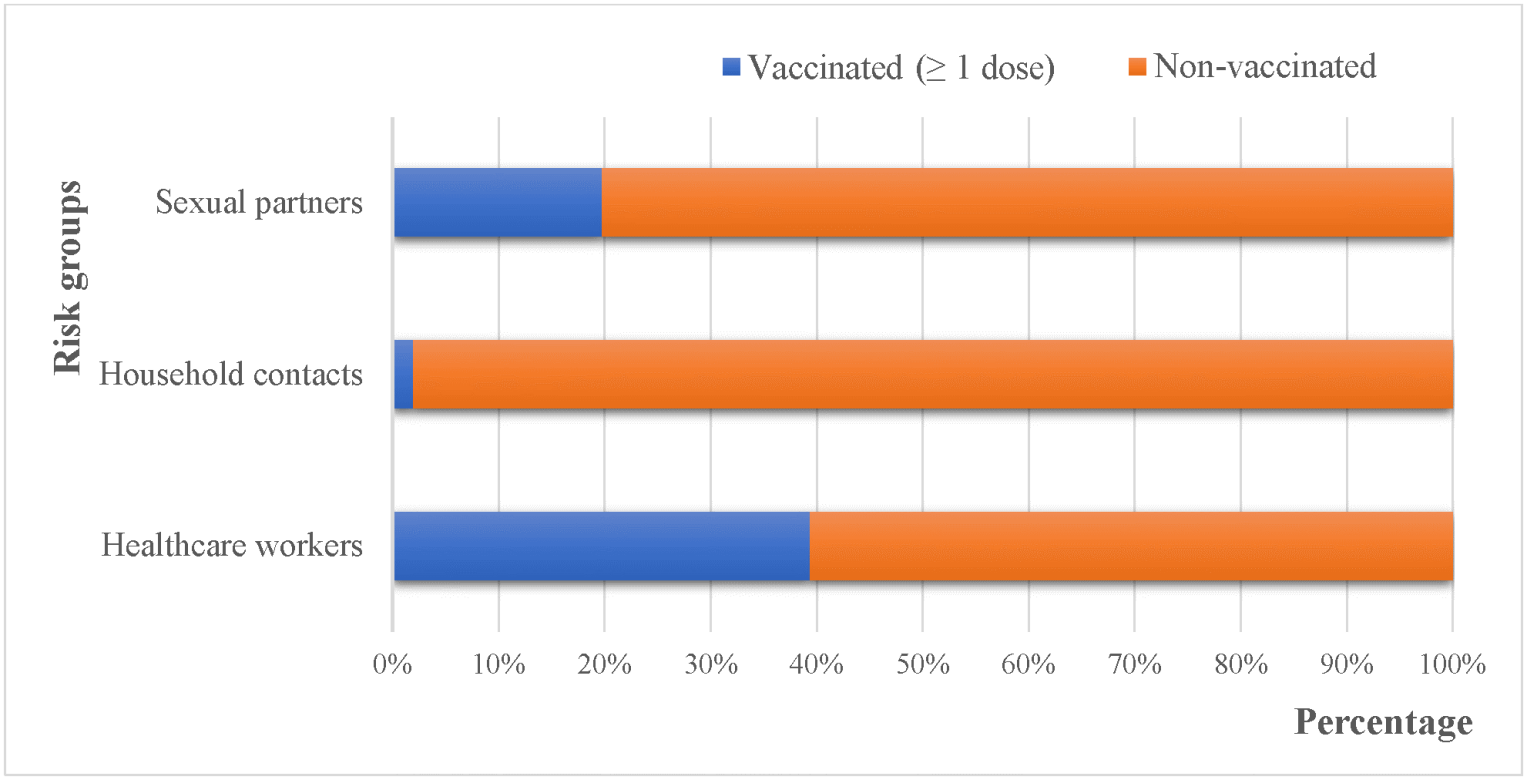
Vaccinated versus non-vaccinated populations in the high-risk groups.

There were more vaccinated HCWs when compared to HHCs and SPs (Fig 1 and Table 2).

**Table 2:** Level of HBV vaccine uptake among the different risk groups (n = 265).

Groups	Vaccinated		Not vaccinated
	≥ 3 doses (Complete)	< 3 doses (Incomplete)	
Healthcare workers, n (%)	47 (37.0)	3 (2.4)	77 (60.6)
Sexual partners, n (%)	6 (8.5)	8 (11.3)	57 (80.2)
Household contacts, n (%)	0 (0)	1 (1.9)	51 (98.1)
Others*	15 (100)	0 (0.0)	0(0.0)
*Total*	68 (25.7)	12 (4.5)	185 (69.8)

*Vaccinated individuals who are neither healthcare workers nor sexual/household contacts of HBV infected patients.

One hundred and eighty-five (69.8%) participants had never received a dose of the vaccine while 68 (25.7%) participants had received at least 3 doses of the vaccine (Table 2). These participants were made up of 44 females and 24 males. Females had a mean age of 33.3 ± 8.6 SD with ages ranging from 19 to 54 years while males had a mean age of 34.2 ± 5.9 with ages ranging from 23 to 49 years. The 185 non-vaccinated cases were made up of 118 females and 67 males and they had a mean age of 31.0 ± 9.0 years.

### Factors affecting vaccine uptake

**Fig 2:**
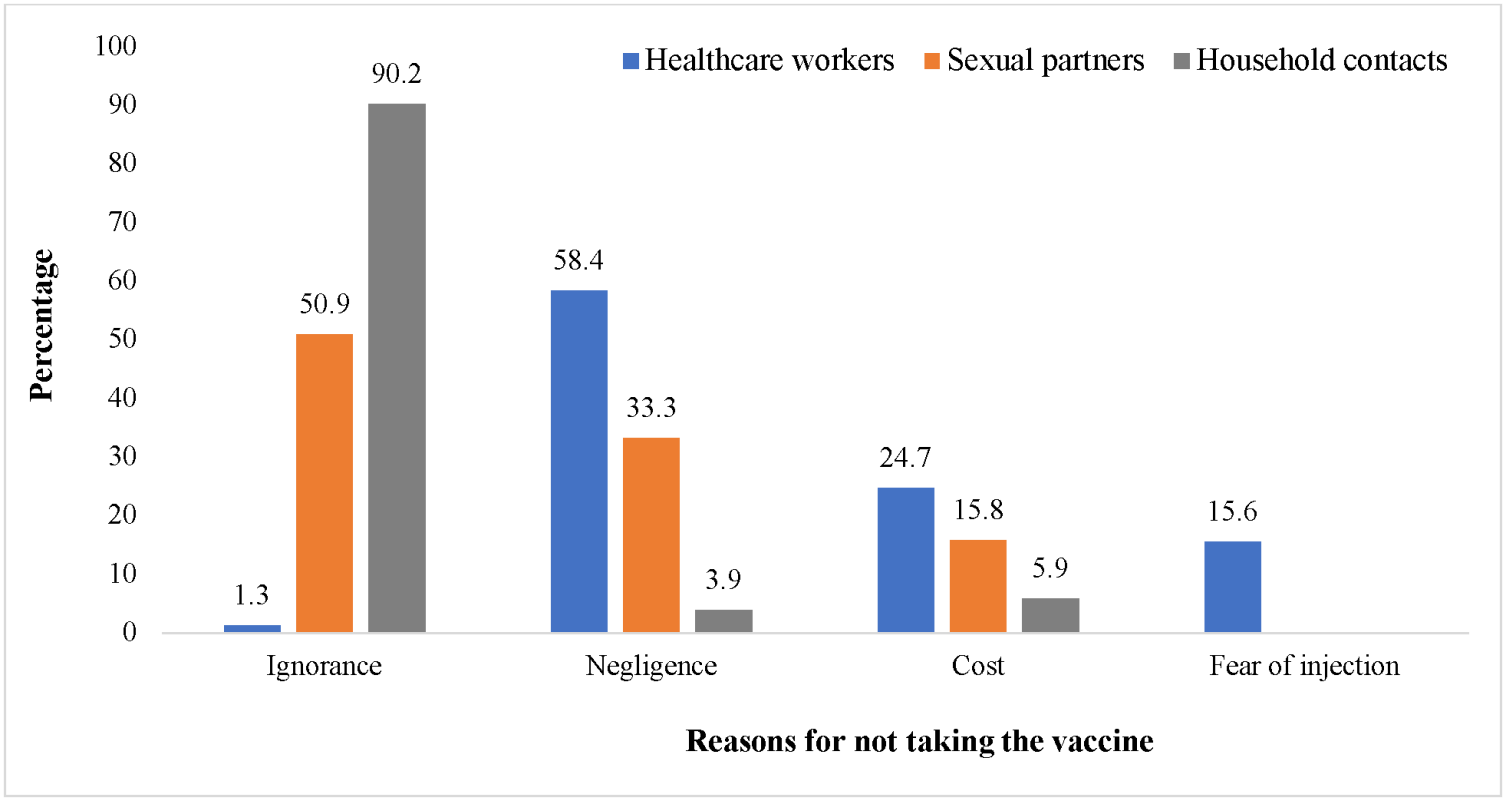
Non-vaccinated individuals and reasons for not taking the vaccine. NB: *Ignorance*: Not being aware of the fact that there is an HBV vaccine. *Negligence*: Aware of the HBV vaccine and its importance yet not vaccinated.

Majority of the household and sexual contacts to HBV infected people had not been vaccinated due to ignorance (Fig 2)

### HBV serological panel results and interpretation

**Fig 3:**
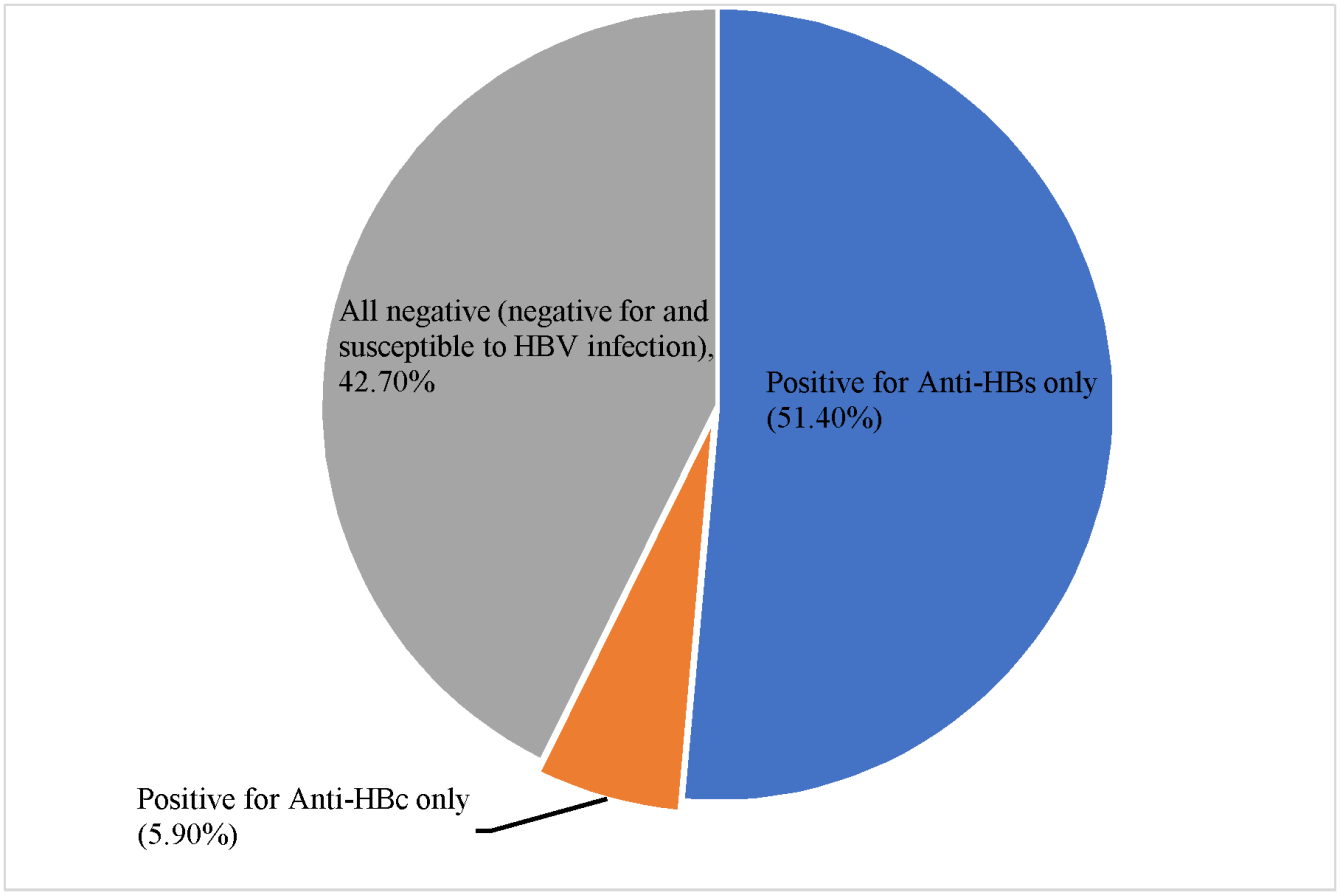
Serological panel results for Participants who have taken ≥ 3 doses of the vaccine (n = 68).

Thirty-five (51.4%) of the vaccinated participants (≥ 3 doses) recorded the expected HBV serologic panel results (positive anti-HBs only) (Fig 3).

**Fig 4:**
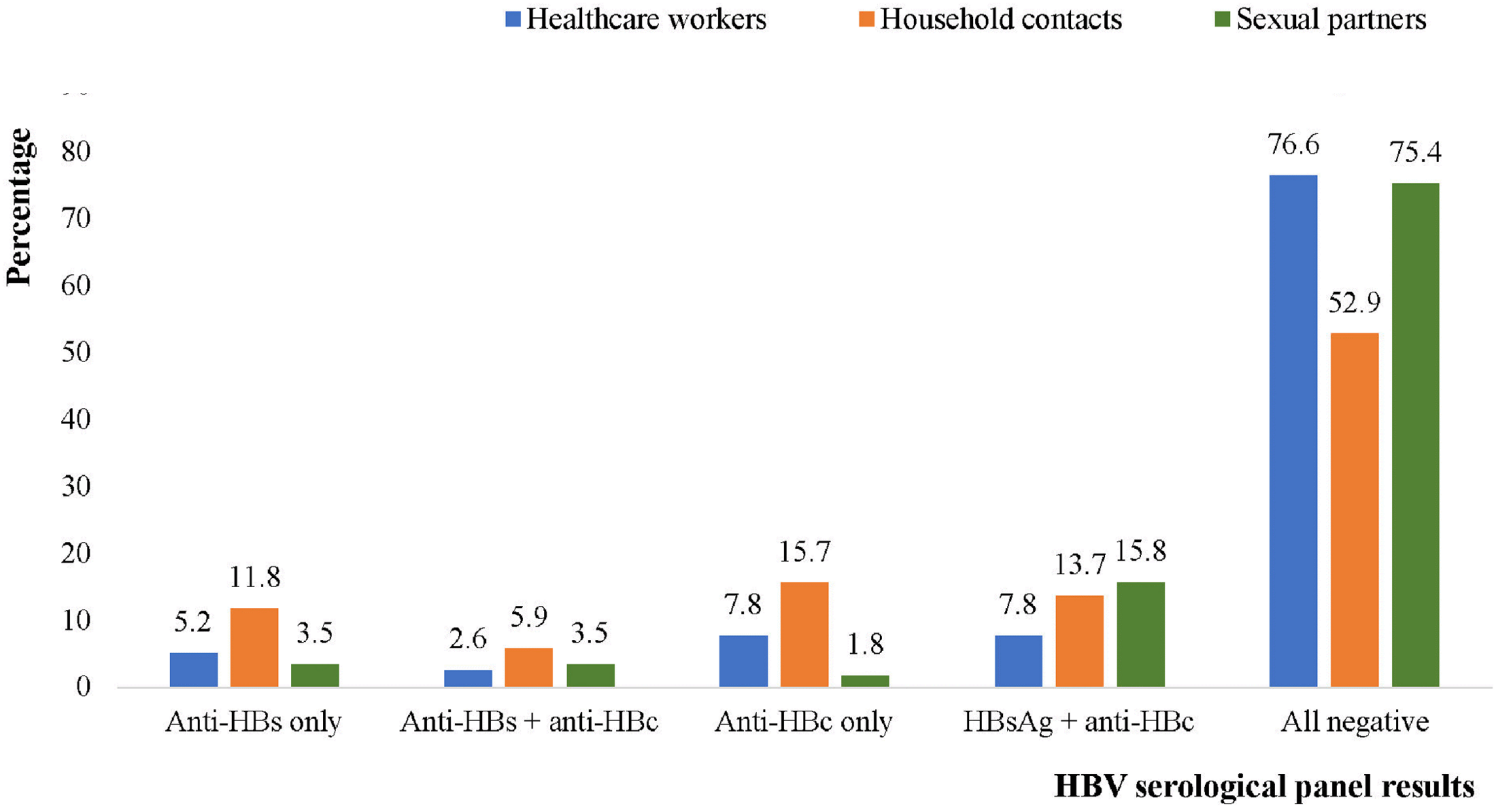
Serological panel results for non-vaccinated participants (n = 185).

Four of the non-vaccinated HHCs (5.2%), 2 SPs (3.5%) and 6 HHCs (11.8%) who had no evidence of past or present HBV infection (negative for HBsAg and Anti-HBc) proved to be positive for anti-HBs only (typical of vaccination) (Fig 4).

### Anti-HBs concentration and factors affecting vaccine response in vaccinated participants who have taken ≥3 doses of the vaccine

**Fig 5:**
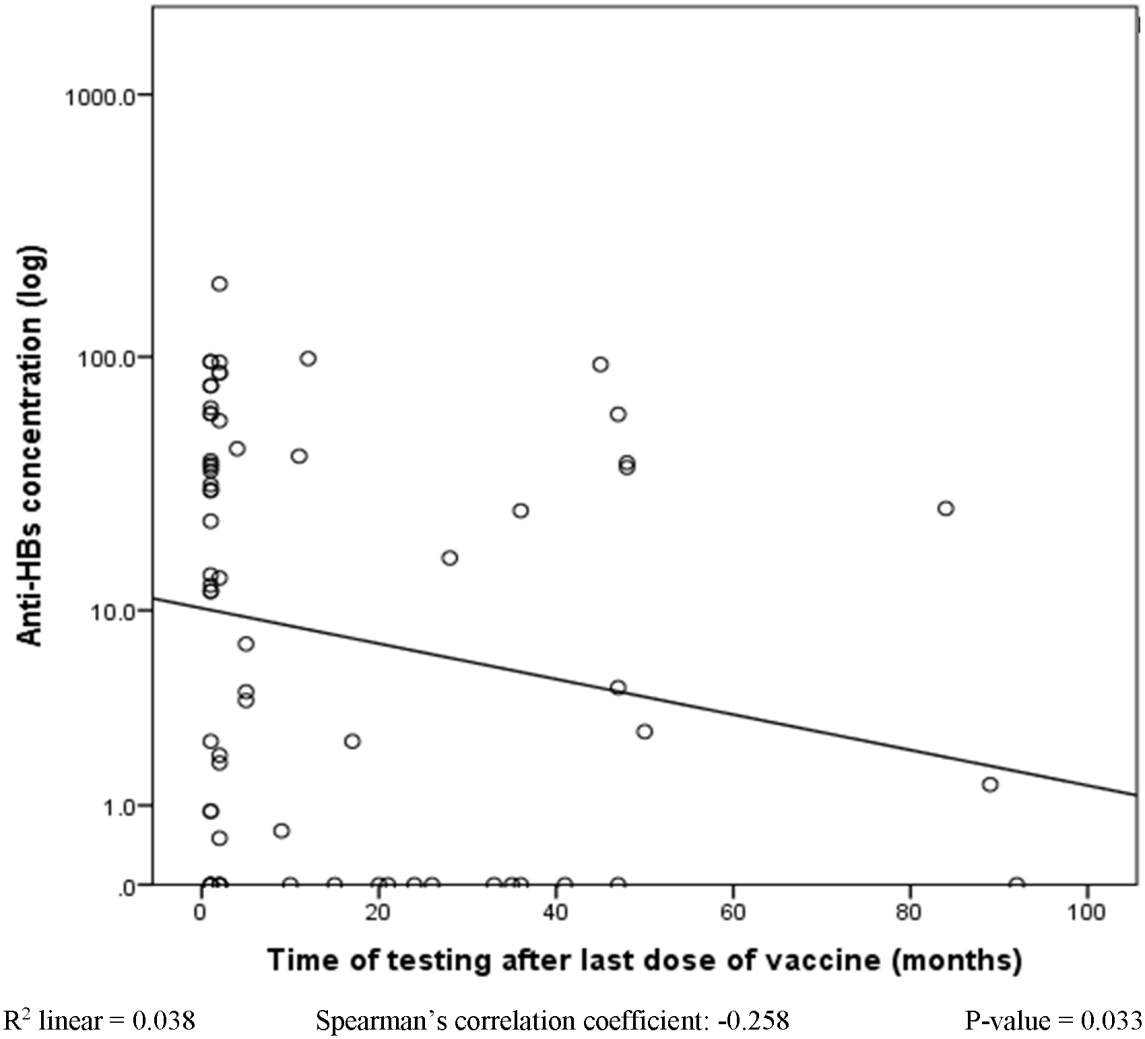
Effect of time on anti-HBs concentration (after 3^rd^ dose of vaccine).

Based on anti-HBs quantitative ELISA, 35 (51.4%) of the vaccinated cases (≥3 dose taken) had anti-HBs concentration ≥10mIU/ml while 33 (33.8%) had anti-HBs concentration < 10mIU/ml. Thirty-seven (54.4%) of the vaccinated cases (≥3 dose taken) had their samples collected and tested within 1–2 months after their 3^rd^ dose while 31 (45.6%) were collected and tested > 2 months after their 3^rd^ dose (the least duration was 4 months while the highest duration was 7 years after the 3^rd^ dose). The participants whose samples were collected 1–2 months after the 3^rd^ dose recorded a significantly higher (p = 0.037, 95% CI: 1.18–36.41) mean anti-HBs concentration (36.46 ± 42.33 mIU/ml) as compared to the mean concentration of those collected >2 months after the 3^rd^ dose (17.66 ± 27.16 mIU/ml) (Table 3). Their difference was as well statistically significant (p = 0.016) when investigating number of cases that had anti-HBs concentration ≥ 10mIU/ml (Table 4). Fig 5 shows decline in anti-HBs concentration over time (4 months to 7 years) for all the 68 vaccinated participants who have received ≥3 doses of the vaccine.

**Table 3:** Summary of Anti-HBs concentrations for vaccinated participants who have received ≥ 3 doses of HBV vaccine (n = 68).

		Time of collection		P-value
		1–2 months after 3^rd^ dose n = 37	> 2 months after 3^rd^ dose n = 31	
**Anti-HBs concentration (mIU/ml)**	Mean ± SD	36.46 ± 42.33	17.66 ± 27.16	**0.037**
	95% CI	22.34–50.57	7.70–27.63	
	Median	23.0	2.8	
	Minimum	0.0	0.0	
	Maximum	190.0	98.5	

**Table 4:** Factors associated with HBV vaccine immune responses among vaccinated participants who had received ≥ 3 doses (n = 68).

Characteristics	Categories	n	Anti-HBs concentration		Risk estimates			
			≥10mIU/ml	<10mIU/ml	Crude Odds ratio (CI)	P-value	Adjusted Odds ratio* (CI)	P-value
Time of sample draw	>2 months after 3^rd^ dose	31	11 (35.5)	20 (64.5)	3.36 (1.24–9.09)	**0.016**	3.45 (1.26–9.47)	**0.016**
	1–2 months after 3^rd^ dose	37	24 (64.9)	13 (35.1)	1		1	
Gender	Male	24	13 (54.2)	11 (45.8)	0.85 (0.31–2.29)	0.743		
	Female	44	22 (50.0)	22 (50.0)	1			
Age group	≥ 40 years	12	6 (50)	6 (50)	1.07 (0.31–3.74)	0.911		
	< 40 years	56	29 (51.8)	27 (48.2)	1			
Alcohol consumption	Yes	46	27 (58.7)	19 (41.3)	0.40 (0.14–1.15)	0.085	0.37 (0.12–1.16)	0.088
	No	22	8 (36.4)	14 (63.6)	1		1	
Smoking	Yes	1	0	1 (100)	3.27 (0.13–83.33)	0.485		
	No	67	35 (52.2)	32 (47.8)	1			
Anti-HBc status	Positive	4	0	4 (100)	10.83 (0.56–209.49)	0.050	10.21(0.16–208.32)	0.110
	Negative	64	35 (54.7)	29 (45.3)	1			
Diabetes	Yes	2	0	2(100)	5.63 (0.26–121.88)	0.232		
	No	66	35 (53.0)	31 (47.0)	1			
Body mass index	≥ 25	43	24 (55.8)	19 (44.2)	0.62 (0.23–1.68)	0.347		
	< 25	25	11 (44.0)	14 (56.0)	1			

*Adjusted for age and sex

P-value from Fisher’s exact test used for all 2 by 2 with cells having values less than 5

Male gender, ≥ 40 years of age, alcohol consumption, smoking and body mass index (BMI) ≥ 25 did not seem to affect the response to the vaccine in our population as none of these factors recorded a statistically significant difference (Tables 4 and 5). Two of the participants where diabetic while 4 where positive for anti-HBc (1 of them was collected 1–2 months after 3^rd^ dose) and all these individuals had anti-HBs concentrations <10mIU/ml after ≥ 3 doses of the vaccine.

**Table 5:** Vaccinated participants collected 1–2 months after 3^rd^ dose and factors affecting vaccine response.

Vaccinated participants collected 1–2 months after 3^rd^ dose (n = 37)		n	Anti-HBs concentration		Risk estimates			
			≥10mIU/ml	<10mIU/ml	Crude Odds ratio (CI)	p P-value	Adjusted Odds ratio* (CI)	P-value
Gender	Male	12	10 (83.3)	2 (16.7)	0.25 (0.05–1.41)	0.103	0.26 (0.05–1.43)	0.120
	Female	25	14 (56.0)	11 (44.0)	1		1	
Age group	≥40 years	5	4 (80)	1 (20)	0.41 (0.04–4.18)	0.638		
	<40 years	32	20 (62.5)	12 (37.5)	1			
Alcohol consumption	Yes	23	17 (73.9)	6 (26.1)	0.35 (0.09–1.43)	0.139	0.53 (0.11–2.41)	0.407
	No	14	7 (50.0)	7 (50.0)	1		1	
Smoking	Yes	1	0	1 (100)	5.88 (0.22–155.08)	0.351		
	No	36	24 (66.7)	12 (33.3)	1			
Anti-HBc status	Positive	1	0	1 (100)	5.88 (0.22–155.08)	0.351		
	Negative	36	24 (66.7)	12 (33.3)	1			
Body mass index	≥25	23	16 (69.6)	7 (30.4)	0.58 (0.15–2.32)	0.443		
	<25	14	8 (57.1)	6 (42.9)	1			

*Adjusted for age and sex

P-value from Fisher’s exact test used for all 2 by 2 with cells having values less than 5

The vaccinated cases in this study took their vaccines from 7 different institutions. None of the institutions provided complete information such as: type of vaccine, date vaccine was received, lot number, manufacturer, route and site of administration of vaccine, funding source and name of vaccinator on the vaccination record of their clients. Four of the institutions were using multi-dose vial vaccines: Euvax (manufactured by LG life science) and/or rDNA HBV vaccine (manufactured by Serum institute of India). None of them respected the ‘not more than 28-day usage instruction’ after opening the vials. They stopped administering the vaccine only upon expiration or when it got finished. One of the institutions did not have any evidence of monitoring the temperature of the fridge where vaccines were stored. Two did not have any contingency plan for vaccine storage in case of power failure or equipment breakdown.

### Anti-HBS concentration in non-vaccinated participants

Non-vaccinated cases had a mean anti-HBs concentration of 3.3 ± 12.2mIU/ml with concentrations ranging from 0 to 85.70 mIU/ml. A total of 19 non-vaccinated participants had anti-HBs concentration ≥10mIU/ml and 166 had concentrations <10mIU/ml.

Twelve (6.5%) non-vaccinated cases tested positive for anti-HBs only with concentrations ≥10mIU/ml. The mean concentration in this group was 37.9 ± 21.2. Seven (3.8%) non-vaccinated cases were positive for anti-HBs and anti-HBc and they all had an anti-HBs concentration ≥10mIU/ml at the time of testing with a mean concentration of 23.9 ±17.5mIU/ml. All the non-vaccinated cases that tested negative for anti-HBs using the serologic panel test had an anti-HBs concentration of 0mIU/ml.

## Discussion

Hepatitis B vaccine provides protection against HBV infection and all its related complications which include chronic hepatitis, fulminant hepatitis, liver cirrhosis and hepatocellular carcinoma (HCC). It happens to be the first vaccine that guarantees protection from a sexually transmitted infection [39].

WHO recommends that everybody belonging to an already identified high-risk group should be vaccinated. Some of these high-risk groups include: people who frequently require blood and/or blood products, dialysis patients, people interned in prisons, intravenous drug users, people with multiple sexual partners, healthcare workers, household and sexual contacts of people with chronic HBV infection. In this study, we assessed the level of vaccine uptake amongst people belonging to three of these high-risk groups: healthcare workers (HCWs), household contacts (HHCs) and sexual partners (SPs) to chronically infected HBV persons.

Our study revealed that amongst all these three high-risk groups, HCWs were the most vaccinated (37.0%). Upon assessing the reasons for not being vaccinated as of the time of enrolment, we realised that majority of the sexual partners and household contacts to chronically infected people had not been vaccinated because of lack of awareness. This was recognised at two different levels: Some of the participants had never heard about hepatitis B infection while others were not aware there was a vaccine against HBV infection. This automatically implies that there is low sensitization coverage on HBV infection in Cameroon despite the high HBV prevalence in the country [5].

Although HCWs recorded the highest number of vaccinated cases amongst the three different high-risk groups, majority of them were still not vaccinated despite their awareness and knowledge on the nature of the infection and its associated complications. Majority of them were just negligent on getting vaccinated while others complained of cost and “fear of injection”. Fear of injection has also been shown to influence vaccine uptake in other studies [40,41]. Nineteen (24.7%) of them complained of cost. Some other studies conducted in different healthcare settings in Cameroon also identified cost as a reason for not taking the vaccine amongst HCWs [36,42].

In our study, a good number of those who complained about the cost of the vaccine admitted that they would love to take the vaccine if the cost is subsidized by the government. The subsidization of HBV vaccine among healthcare workers is really an issue worth addressing in Cameroon considering the fact that the vaccine was included in the national Expanded Programme for immunization (EPI) since 2005 and its now given free of charge to children. In this same light, the healthcare system can as well subsidize the vaccine for HCWs bearing in mind that the nature of their profession practically exposes them to the infection.

Several studies assessing vaccine uptake among healthcare workers in Cameroon (conducted between 2013 and 2016) revealed a low percentage uptake ranging from 12.3% to 24.5% [36,42–44] as compared to our findings (37.0%). However, the higher vaccine uptake in our study compared to others conducted in Cameroon may not be due to fact that participants in our study were more health conscious than their counterparts in previous studies. We can attribute this high uptake to some form policy obligation. Most of the HCWs enrolled in this study were from the Buea Regional Hospital (BRH) of the South West Region of Cameroon. The Laboratory Department of BRH got enrolled into an international accreditation program in 2010 which recommended all laboratory personnel to be vaccinated against HBV infection. As such, a good proportion of them subsequently got vaccinated. Similarly, as a recommendation to work in the BRH haemodialysis unit at its creation, all staff were advised to take the HBV vaccine and the program incurred the cost. These internal policies improved the number of vaccinated HCWs in our study population. Therefore, hospital administrations in Cameroon can improve on the vaccine uptake of HCWs if they increase the awareness campaigns in their respective establishments and urge or oblige personnel to get vaccinated. The cost of the vaccine could also be subsidised at the level of the hospital administration to encourage vaccine uptake amongst HCWs. Previous studies conducted out of Cameroon have recorded higher levels of vaccine uptake among HCWs [45–48]

The proportion of non-vaccinated cases in this study was quite significant and they recorded varying HBV serological profile results. Interestingly, 12 (6.5%) of the non-vaccinated cases had results typical of what is expected for vaccinated cases (positive anti-HBs only). Few studies have actually reported such relatively common phenomenon in unvaccinated HCWs [49–51]. Studies carried out among healthcare workers in 1985 revealed a very significant proportion of HCWs with isolated anti-HBs [50,51]. Some researchers believe that this probably represents a nonspecific antibody response to a cross reacting antigen rather than immunization with hepatitis B [51]. In addition to representing vaccination, an isolated anti-HBs may be attributed to low level exposure to blood containing HBsAg only and not the intact virus [49]. In the blood of HBV infected individuals, HBsAg is usually found on the surface of intact viruses or secreted as independent lipoprotein particles. The HBsAg independent lipoprotein particles in blood is usually in excess as compared to intact viruses in the ratio of 1000:1 [31] so there is a high chance that an uninfected person may come in contact with a small amount of infected blood that contains mainly the HBsAg non-infectious lipoprotein particles. For such situations, the individual at risk may likely develop only anti-HBs in response to the HBsAg particles. Anti-HBc can only be produced when an individual comes in contact with the intact virus that contains the HBcAg (Hepatitis B-core antigen) and HBV DNA material. With this in mind, testing for the presence of HBsAg, anti-HBc or anti-HBs may all be helpful in determining possible exposure to HBV infected fluid. The 12 non-vaccinated participants with isolated anti-HBs all had anti-HBs concentrations ≥ 10mIU/ml indicative of immunity to HBV infection. In this context, one may consider this as some form of naturally acquired immunity to HBV infection even in the absence of anti-HBc.

Presence of anti-HBs and anti-HBc is the most common serological pattern usually seen in individuals who have naturally acquired immunity to HBV infection [30]. Previous studies have reported the occurrence of naturally acquired immune responses to HBV in high-risk groups [52,53]. In line with these studies, 9 of such cases were recorded in our study: 2 vaccinated and 7 non-vaccinated cases, with anti-HBs concentrations ≥ 10mIU/ml.

There may be cases where an “isolated anti-HBc” would be the only detectable marker [30]. Such cases usually have more than one possible clinical interpretation which include: distant resolved HBV infection (the most common interpretation); false anti-HBc positive result (more common in people living in a non-endemic area), resolving acute HBV infection (in the period between HBsAg loss and development of detectable anti-HBs), passive transfer of maternal anti-HBc (in children up to 3 years of age) and occult HBV infection (where HBV DNA is detectable in the absence of HBsAg). This can be a chronic antigen carrier state where the individual either does not produce or produces undetectably low levels of HBsAg [31]. We identified 19 participants with “isolated anti-HBc” in this study: 15 non-vaccinated and 4 vaccinated participants. All of them had an anti-HBs concentration of 0mIU/ml. Because these participants all belong to high-risk groups in an HBV endemic setting, there is a high possibility that the isolated anti-HBc results were not false negative results [54]. However, because we did not assess HBV DNA levels in these participants, we cannot conclude that these were occult HBV cases. The total absence of anti-HBs (0mIU/ml) for all these cases (even among vaccinated cases with isolated anti-HBc) may indicate no evidence of resolution of infection whether past or recent.

All the participants who had a positive anti-HBs result with the HBV serological panel recorded anti-HBs concentrations ≥10mIU/ml. Individuals who attain anti-HBs levels of ≥10 mIU/mL within 1–2 months after having received ≥3 dose of HBV vaccine series are considered as vaccine responders or immune to HBV infection. Our study showed that the number of vaccinated cases with anti-HBs ≥10 mIU/mL whose samples were collected within 1–2 months after their 3^rd^ dose was significantly higher than the number of vaccinated cases with anti-HBs ≥10 mIU/ml whose samples were collected >2 months after their 3^rd^ vaccine dose. This justifies the fact that anti-HBs concentrations decline over time as seen in other studies [17,55]. However, it does not imply that vaccine protection is short lived because immunological memory cells persist [56] and this can guarantee protection for ≥22 years [6,17].

Over 90% of individuals who took ≥3 dose of HBV vaccine attain the desired anti-HBs concentration (≥10 mIU/mL) within 1–2 months after the last dose [17,57]. We measured anti-HBs levels for 37 vaccinated participants (took ≥3 doses) 1–2 months after their last dose and only 24 (64.9%) had anti-HBs levels ≥10 mIU/mL. This proportion is far lower than the 90% of responders described by other researchers. It actually implies that we may have a significant proportion of vaccinated individuals in our community who believe that they are immune to HBV infection whereas they are not. An individual who thinks he is protected meanwhile he is not may seemingly be more at risk of contracting the infection as compared to somebody who knows he is not protected. This is because a non-protected person who thinks he is protected may likely unhesitatingly engage himself/herself into some risky activities without taking any precaution. Our findings further stresses on the importance of a post vaccination test for anti-HBs within 1–2 months after the last vaccine dose [17,30] and this should be considered for all vaccinated individuals and not only people belonging to high-risk groups.

A vaccine response as low as 64.9% in a study population should probably trigger an investigation to identify the possible risk factors that might have led to poor response. We assessed the role of some already established risk factors and none of them were significantly associated with poor response in our study population. However, we had 2 diabetic cases, 4 anti-HBc positive cases and 1 smoker who all had anti-HBs levels <10mIU/ml. Our study did not have any HIV infected vaccinated person nor any vaccinated hemodialysis patient. Unfortunately, the role of genetic factors could not be assessed in this study.

Another issue worth questioning here is the potency of the vaccine administered. Unfortunately, we could only visit 4 out of the 7 recognized institutions that administered vaccines to our participants. All the four were using multi-dose vials vaccine and none of them discarded or stopped using the content of the vials after 28 days of usage (counting the day it was opened as day 1) as recommended by WHO [58] or as indicated on the package insert of the vaccines. They only stopped using the vaccine when it finished or when it got expired. One of the institutions did not have evidence of monitoring the temperature of the fridge were their vaccines were stored and 2 did not have any reliable contingency plan in case of power failure or equipment (fridge) breakdown despite the constant power failure and poor service maintenance of equipment in the study area. The cost of the vaccine ranged from 2,500 XAF per dose to 10,000 XAF per dose in the 7 different institutions. We could not establish why there was much difference in the cost prices of the vaccines across institutions, but this could imply in one way or another that they had different suppliers with products from unrelated sources. More research needs to be conducted to properly assess the influence of some of these factors on the potency of the vaccine. We also realized that no standardized method of documenting vaccination records was in place. Not all the information recommended by CDC [59] was found on the vaccination card of participants. All these findings can easily make one to question the competency of those administering vaccines as well as the potency of the vaccines.

In conclusion, vaccine uptake was best among healthcare workers as compared to all the other high-risk groups in this study although the proportion of vaccinated healthcare workers was still not good enough. Most studies that have addressed vaccine uptake and efficacy worked with healthcare workers only but we assessed household contacts as well as sexual partners to HBV infected persons. Sensitization on the importance of HBV vaccine really needs to be intensified among high-risk groups so that some recurrent reasons for not taking the vaccine like ignorance and negligence can be eliminated. The cost of taking the vaccine still stands as a major barrier to vaccine uptake in our study population. It could be of paramount importance to identify people who are already naturally immune to HBV prior to vaccination because such people would most likely not need to take the vaccine and this would limit their expenditure. The health sector should standardize the protocol for administering vaccines, train vaccine administrators, encourage vaccination via intensive sensitization, subsidize the cost of taking the vaccine among healthcare workers and encourage anti-HBs post vaccination testing among all vaccinated individuals.

## Supporting information

S1 File Questionnaire. (PDF)

S2 File Raw data files. (XLSX)

## Abbreviations

Anti-HBc (anti-HBc): Hepatitis B core antibody
Anti-HBs: Hepatitis B surface antibody
CDC: Center for disease control and prevention
EPI: Expanded Programme for immunization
HBsAg: Hepatitis B surface antigen
HBV: Hepatitis B virus
HCV: Hepatitis C virus
HCW: Healthcare worker
HHC: Household contact
HIV: Human Immunodeficiency virus
NECRHH: National Ethics Committee of Research for Human Health
SP: Sexual partner
SPSS: Statistical Package for Social Sciences
WHO: World Health Organisation
