# Supplementary material for: Vaccine uptake and immune responses to HBV infection amongst vaccinated and non-vaccinated healthcare workers, household and sexual contacts to chronically infected HBV individuals in the South West Region of Cameroon
Source: PLoS One. 2018 Jul 16;13(7):e0200157. doi: 10.1371/journal.pone.0200157 (PMC6047772; doi:10.1371/journal.pone.0200157)
Supplement: S1 File — (PDF) [file pone.0200157.s001.pdf]

RESEARCH QUESTIONNAIRE

Virologic, serologic, hematologic and biochemical characterization of hepatitis B virus infection in North West and South West regions of Cameroon: transmission risk and immune responses in the subjects

PATIENT IDENTIFICATION: Index case ☐ Sexual Partner ☐ Household contact ☐ Healthcare worker ☐ Healthy control ☐

1. **Participant code:** ____________________ **Partner N⁰:** ______________ Date of collection**:** ______________________________

Address: ____ Tel:____

DEMOGRAPHIC/SOCIO-ECONOMIC STATUS

2. Date of birth: ______________________ OR Age: _________________ Ethnic Origin: ____
3. Religion: Muslim ☐ Christians ☐ If Christian which dominion? Catholic ☐ Protestant ☐ Revival ☐
4. Gender (tick one box only): Male ☐ Female ☐ If female: are you pregnant? No ☐ Yes ☐
5. Occupation:_______________________ Civil servant ☐ Employed in private the sector ☐ Self employed ☐
6. Monthly Income: < 50,000F ☐ 50,000-100,000F ☐ >100,000F ☐ None ☐
7. Level of Education: Did not go to school ☐ Primary ☐ Secondary ☐ Post-secondary ☐
8. Marital status: Single ☐ Currently married ☐ Previously married (Divorced/widowed) ☐
9. If "currently married", type of marriage: Monogamy ☐ Polygamy ☐

KNOWLEGDE ON HEPATITIS B/ VACCINE STATUS

10. Have you heard about Hepatitis B before? Yes ☐ No ☐
11. How is Hepaitis B transmitted? Through drinking water/food ☐ Via contact with infected body fluids ☐ Having unprotected sex with an infected person ☐ Airborne ☐ By sharing cups and plates with an infected person ☐ Through blood transfusion with an infected blood ☐ By sharing blades and other sharp objects with an infected person ☐
13. How can Hepatiis B be prevented? By taking the vaccine ☐ By always covering the mouth before coughing ☐ By having protected sex ☐ By keeping the environment clean ☐
14. Have you been vaccinated for Hepatitis B? Yes ☐ No ☐ Never heard of HBV vaccine ☐ If No, why ?___________________
15. If yes, how many doses did you receive? ____________ Don't know ☐
16. Have you been tested for Hepatitis? Yes ☐ No ☐ If yes which of the hepatitis? HBV ☐ HCV ☐
17. Reasons for being tested for Hepatitis: Voluntary ☐ Administrative ☐ Antenatal ☐ STI consultation ☐ Confirmation ☐ Routine ☐ Blood donation ☐ Signs and symptoms __________________
18. What were the results? HBV: Positive ☐ Negative ☐ HCV: Positive ☐ Negative ☐ If "positive", at what age? ______
19. If positive, are you on any treatment? Yes ☐ No ☐
20. Type of treatment: traditional ☐ antiviral ☐ Immune boosters ☐

HEPATITIS/ RISK FACTORS

18. Age at first sexual intercourse:_________ Gender of sexual partners: Men ☐ Women ☐ Both ☐
19. How often do you use condoms during sexual intercourse? Never ☐ Rarely ☐ Sometimes ☐ Often ☐ Usually ☐
20. How many sexual partners have you had in the last 12 months? _________ in the How many sexual partners have you had in your lifetime?___________ Do you know the status of your partner ? ______ What is it? _____
18. Have you ever been diagnosed with a sexually transmitted disease in the past? Yes ☐ No ☐
19. Have you ever used injection drugs? Yes ☐ No ☐ If yes, for how long did you use injection drugs? _______________
20. Do you currently use injection drugs? Yes ☐ No ☐ If yes, about how many times over the past month: _____________
21. Have you had known contact with an individual with Hepatitis B or C? Yes ☐ No ☐
22. If yes, were they: Sexual partner ☐ Household member ☐ Occupational contact ☐ Injection drug user ☐

What is the nature of your relationship with household member (s) ________________________

23. Have you ever had a blood transfusion? Yes ☐ No If yes, how long ago?___________________
24. Do you have a tatoo on your body? Yes ☐ No ☐

25. Do you usually ensure that the barber disinfects his shaving machine before using it on you? Yes ☐ No ☐

26. Nature of Secondary school attended: Boarding ☐ Days school ☐ Both ☐

27. Have you been incarcerated before? Yes ☐ No ☐ If "yes" for how long? ___________________________

28. Do you currently smoke? Yes ☐ No ☐ or ex-smoker ☐ If "Yes" for how long have been smoking? ___________

29. How many stick of cigarette do you smoke per day? ________________ Do you smoke substances other than cigarette? Yes ☐ No ☐

30. Do you currently consume alcohol? Yes ☐ No ☐ Ex-alcohol consumer ☐

31. **Alcohol use disorder identification (AUDIT)**: Place a tick in the box that best describe your answer to the question (**alcohol consumers only)**

Questions	0	1	3	3	4	score
How often do you have a drinks containing alcohol?	Never	Monthly or less	2 to 4 times a month	2 to 3 times a week	4 or more times a week	
How many drinks do have containing alcohol on a typical day you are drinking?	1 or 2	3 or 4	5 or 6	7 to 9	10 or more	
How often do you have 5 or more alcoholic drinks on one occasion?	Never	Less than monthly	Monthly	Weekly	Daily or almost daily	
How often during the last year, have you found that you were able to stop drinking once you started?	Never	Less than monthly	Monthly	Weekly	Daily or almost daily	
How often during the last year, have you failed to do what was normally expected of you because of drinking?	Never	Less than monthly	monthly	Weekly	Daily or almost daily	
How often during the last year have you needed a first drink in the morning to get yourself going after a heavy drinking session?	Never	Less than monthly	Monthly	Weekly	Daily or almost daily	
How often during the last year, have you had a feeling of guilt or remorse after drinking?	Never	Less than monthly	Monthly	Weekly	Daily or almost daily	
How often during the last year, have you been unable to remember what happened the night before because of your drinking?	never	Less than monthly	Monthly	Weekly	Daily or almost daily	
Have you or someone else been injured because of your drinking?	Never	Less than monthly	Monthly	Weekly	Daily or almost daily	
Has a relative, friend, doctor or other health care worker been concerned about your drinking or suggested you cut down?	never	Less than monthly	Monthly	Weekly	Daily or almost daily	
**Total score**						

Conclusion: Hazardous drinking (**score ≥ 8**) ☐ non-hazardous drinking (**score less than < 8**) ☐

32. Other comorbodities: Diabetes ☐ Hypertensive ☐ Liver diseases ☐ Kidney disease ☐

33. For how long have you been suffering from this other comobidities?______________

CLINCAL EVALUATION

At enrollment

Transaminases level (IU/ml) AST:________________ ALT level: ___________________ AST/ALT ratio _____________ APRI score ___________________

HBV profile panel: HBsAG ____________HBsAb_____________ HBeAg ___________ HBeAb __________________ HBV total core Ab: _____________

HBV DNA level: _____________________ (copies/mL) HBV genotype: ____________________________ Age platelet ratio_________________

Mass: ____________________________ Height_________________ Signs and symptoms: ____________________________

At follow-up

Transaminases level (IU/ml) AST:________________ ALT level: ___________________ AST/ALT ratio _____________ APRI score ___________________

HBV profile panel: HBsAG ____________ HBsAb_____________ HBeAg ___________ HBeAb __________________ HBV total core Ab: _____________

HBV DNA level: _____________________ (copies/mL) HBV genotype: ____________________________ Age platelet ratio_________________

Mass: ____________________________ Height_________________ Signs and symptoms: ____________________________

We immensely appreciate your participation!
